# Supplementary material for: Health facility availability and readiness for family planning and maternity and neonatal care services in Nepal: Analysis of cross-sectional survey data
Source: PLoS One. 2023 Aug 7;18(8):e0289443. doi: 10.1371/journal.pone.0289443 (PMC10406287; doi:10.1371/journal.pone.0289443)
Supplement: S1 Fig — (DOCX) [file pone.0289443.s002.docx]

**S1 Fig. Types of contraceptives provided according to background characteristics of the health facilities in 2015 and 2021**


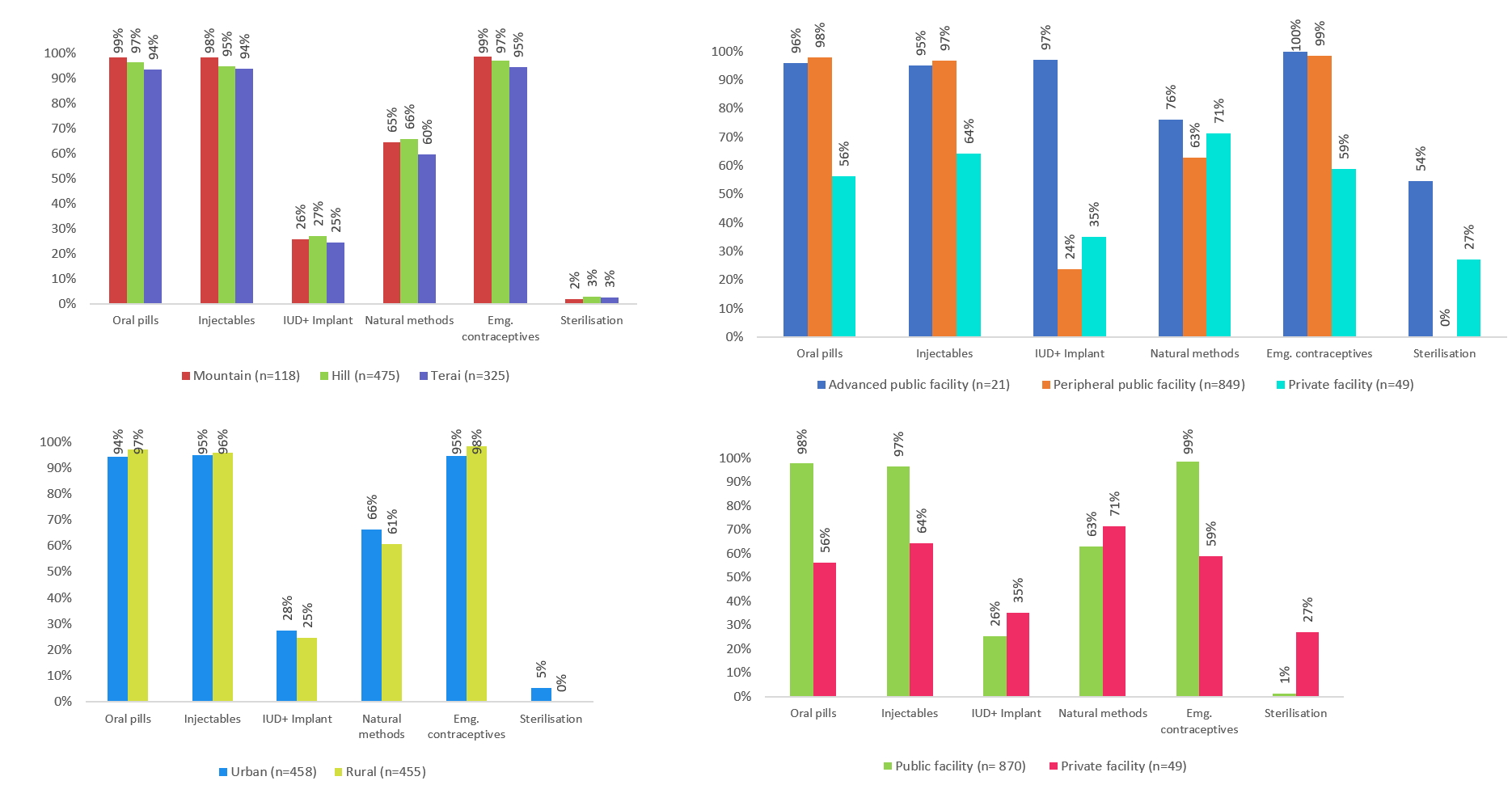


1. NHFS 2015


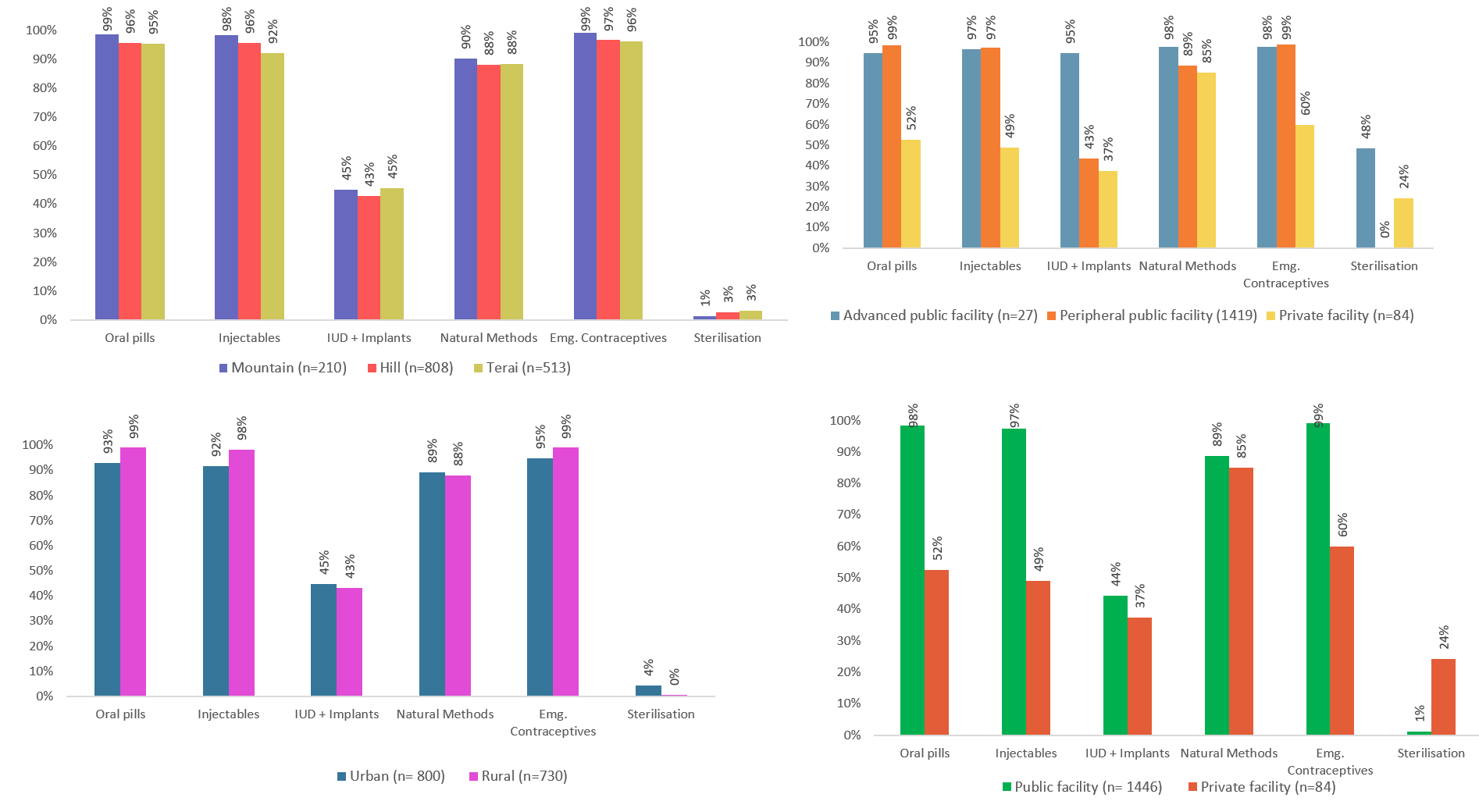


(b) NHFS 2021

*Emergency contraceptives include emergency contraceptives pills, condom, (also includes spermicide and diaphragm in 2021), Oral contraceptives include combined oral pills and progestin only pills, Permanent sterilisation includes both male and female sterilisation, Natural methods include counselling on natural methods broadly in 2015, but in 2021, it also includes data on lactational amenorrhea, counselling on withdrawal method, beads for standard days methods.
