# Supplementary material for: Health facility availability and readiness for family planning and maternity and neonatal care services in Nepal: Analysis of cross-sectional survey data
Source: PLoS One. 2023 Aug 7;18(8):e0289443. doi: 10.1371/journal.pone.0289443 (PMC10406287; doi:10.1371/journal.pone.0289443)
Supplement: S2 Fig — (DOCX) [file pone.0289443.s003.docx]

**S2 Fig. Availability of basic emergency obstetric and newborn care signal functions in 2015 and 2021 according to background characteristics of the health facilities**


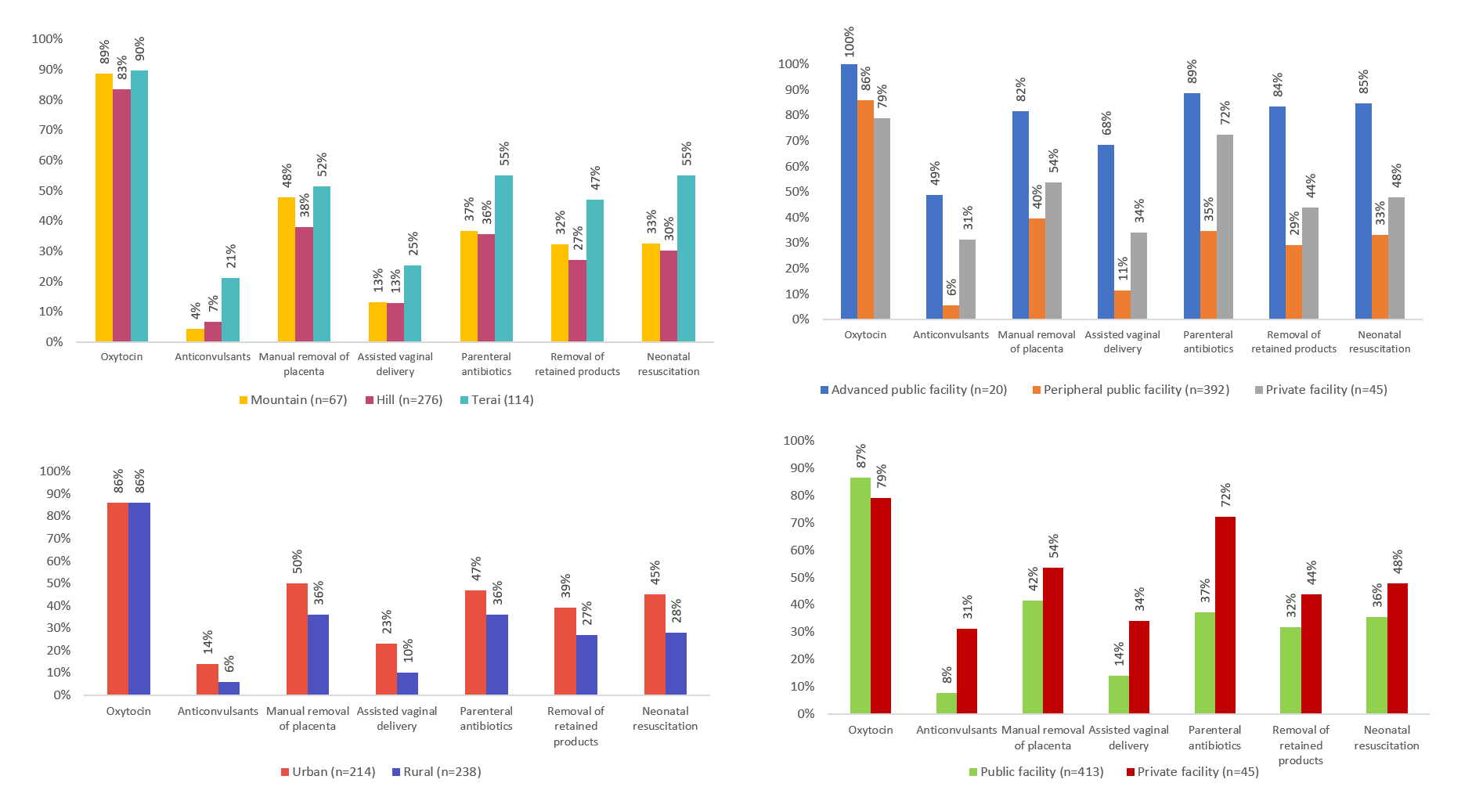


1. NHFS 2015


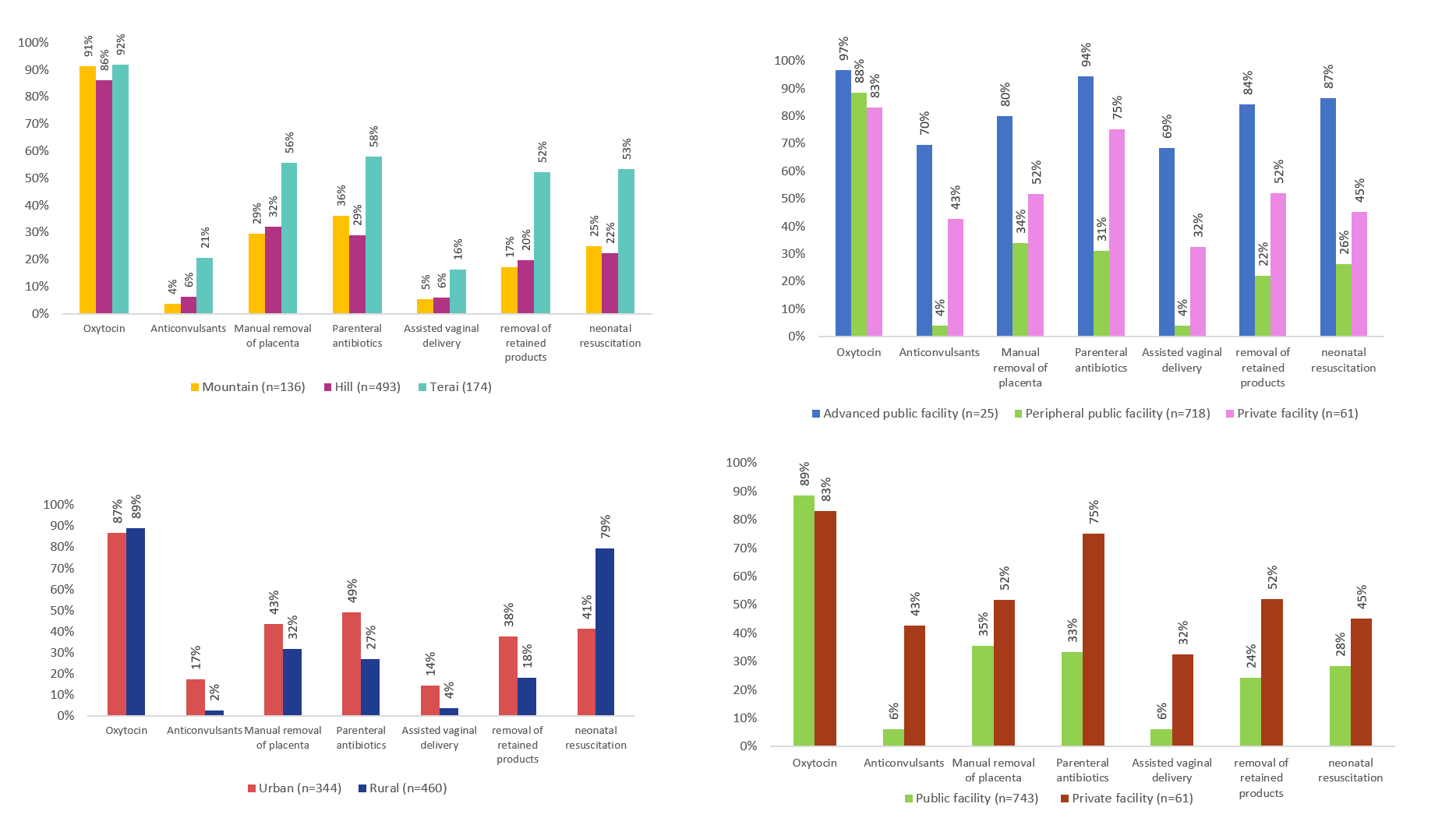


1. NHFS 2021
