# Supplementary material for: Health facility availability and readiness for family planning and maternity and neonatal care services in Nepal: Analysis of cross-sectional survey data
Source: PLoS One. 2023 Aug 7;18(8):e0289443. doi: 10.1371/journal.pone.0289443 (PMC10406287; doi:10.1371/journal.pone.0289443)
Supplement: S1 Table — (DOCX) [file pone.0289443.s004.docx]

**S1 Table. Tracer items used in the calculation of service specific readiness indexes**

| **Family Planning service readiness index** | | | | | |
| --- | --- | --- | --- | --- | --- |
| Domains | Tracer items | Yes | No | Max. Total | Percent  weight (%) |
| Staff and guidelines | Guidelines for diagnosis and treatment of FP | 1 | 0 | 2 | 33.3 |
|  | At least one staff member trained in FP | 1 | 0 |  |  |
| Equipment | Blood pressure (BP) apparatus | 1 | 0 | 1 | 33.3 |
| Medicines and commodities | Combined estrogen progesterone  oral contraceptive pills | 1 | 0 | 3 | 33.3 |
|  | Injectable contraceptives | 1 | 0 |  |  |
|  | Condoms | 1 | 0 |  |  |
| **Antenatal care domains and readiness index** | | | | | |
| Domains | Tracer items | Yes | No | Max. Total | Percent weight (%) |
| Staff and guidelines | Guidelines on antenatal care | 1 | 0 | 2 | 25 |
|  | Staff trained in antenatal care | 1 | 0 |  |  |
| Equipment | Blood pressure apparatus | 1 | 0 | 1 | 25 |
| Diagnostics | Haemoglobin | 1 | 0 | 2 | 25 |
|  | Urine dipstick - protein | 1 | 0 |  |  |
| Medicines and commodities | Iron and folic acid combined tablets | 1 | 0 | 3 | 25 |
|  | Albendazole | 1 | 0 |  |  |
|  | Tetanus toxoid vaccine | 1 | 0 |  |  |
| **Basic emergency obstetric and newborn care (BEmONC) readiness index** | | | | | |
| Domains | Tracer items | Yes | No | Max total | Percent weight (%) |
| Staff and guidelines | Guidelines for delivery and newborn care | 1 | 0 | 2 | 33.3 |
|  | Staff trained in delivery and newborn care | 1 | 0 |  |  |
| Equipment | Emergency transport | 1 | 0 | 14 | 33.33 |
|  | Sterilisation equipment | 1 | 0 |  |  |
|  | Examination light | 1 | 0 |  |  |
|  | Delivery pack | 1 | 0 |  |  |
|  | Suction apparatus | 1 | 0 |  |  |
|  | Manual vacuum extractor | 1 | 0 |  |  |
|  | Vacuum aspirator or D&C kit (with speculum) | 1 | 0 |  |  |
|  | Neonatal bag and mask | 1 | 0 |  |  |
|  | Delivery bed | 1 | 0 |  |  |
|  | Partograph | 1 | 0 |  |  |
|  | Gloves | 1 | 0 |  |  |
|  | Infant weighting scale | 1 | 0 |  |  |
|  | Blood pressure apparatus | 1 | 0 |  |  |
|  | Soap and running water or alcohol-based hand rub | 1 | 0 |  |  |
| Medicines and commodities | Antibiotic eye ointment for newborn | 1 | 0 | 6 | 33.33 |
|  | Injectable uterotonic | 1 | 0 |  |  |
|  | Injectable antibiotic | 1 | 0 |  |  |
|  | Magnesium sulphate | 1 | 0 |  |  |
|  | Skin disinfectant | 1 | 0 |  |  |
|  | Intravenous solution with infusion set | 1 | 0 |  |  |

Example showing how the readiness score was calculated for a health facility with only one tracer item for the domain of ‘Staff and guidelines’, one tracer item for equipment and two tracer items for the domain ‘Medicines and commodities’:

Readiness Index score:


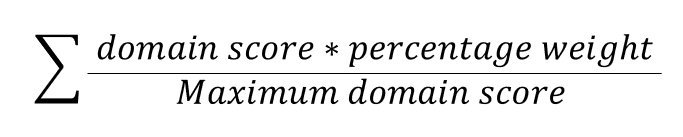


= 1*33.33/2 + 1*33.33/1 + 2*33.33/3

= 16.67+33.33+22.22

= 72.22% or 0.72
