# Supplementary material for: Health facility availability and readiness for family planning and maternity and neonatal care services in Nepal: Analysis of cross-sectional survey data
Source: PLoS One. 2023 Aug 7;18(8):e0289443. doi: 10.1371/journal.pone.0289443 (PMC10406287; doi:10.1371/journal.pone.0289443)
Supplement: S2 Table — (DOCX) [file pone.0289443.s005.docx]

**S2 Table. Presence of tracer items for family planning, antenatal care and basic emergency obstetric and newborn care services 2015 vs 2021**

| **Family planning domains** | Tracer items | Facilities with tracer items, n (%) | |
| --- | --- | --- | --- |
|  |  | NHFS 2015 (n=919) | NHFS 2021 (n=1530) |
| Staff and guidelines | National family planning guidelines* | 118(12.5) | 312 (20.4) |
|  | Trained family planning Provider | 287(30.6) | 320 (20.9) |
| Equipment | Blood pressure (BP) apparatus | 796(84.6) | 1474 (96.4) |
| Medicines and commodities | Combined estrogen progesterone  oral contraceptive pills | 874(95.1) | 1461 (95.5) |
|  | Injectable contraceptives | 873(95) | 1451 (94.8) |
|  | Condoms | 887(96.5) | 1481 (96.8) |
| **Antenatal care domains** | Tracer items | Facilities with tracer items, n (%) | |
|  |  | NHFS 2015 (n=920) | NHFS 2021(n=1538) |
| Staff and guidelines | ANC guidelines** | 230(25) | 161(10.5) |
|  | Trained ANC Provider | 247(26.9) | 424(27.6) |
| Equipment | Blood pressure (BP) apparatus | 790(85.9) | 1498(97.4) |
| Diagnostics | Haemoglobin | 137(14.9) | 390(25.3) |
|  | Urine Protein*** | 140(15.2) | 434(28.2) |
| Medicines and commodities | Combined folic acid and iron tables | 835(90.8) | 1471(95.6) |
|  | Tetanus toxoid | 232(25.3) | 469(30.5) |
|  | Albendazole | 898(97.6) | 1440(93.6) |
| **Basic emergency obstetric and newborn care domains** | Tracer items | Facilities with tracer items, n (%) | |
|  |  | NHFS 2015 (n=457) | NHFS 2021 (n=804) |
| Staff and guidelines | National guidelines for childbirth | 100(21.8) | 103(12.8) |
|  | Trained delivery Provider | 148(32.5) | 219(27.3) |
| Equipment | Blood pressure (BP) apparatus | 379(82.9) | 762(94.8) |
|  | Examination light | 278(60.7) | 754(93.8) |
|  | Delivery pack | 419(91.7) | 786(97.7) |
|  | Delivery bed | 441(96.3) | 794(98.7) |
|  | Blank partograph | 366(80.0) | 727(90.4) |
|  | Suction apparatus for newborn | 283(62.0) | 528(65.7) |
|  | Manual Vacuum extractor | 95(20.7) | 187(23.2) |
|  | Vacuum aspirator or MVA kit | 88(19.2) | 168(20.9) |
|  | Sterilisation equipment | 436(95.3) | 804(100) |
|  | Neonatal bag and mask | 379(82.8) | 737(91.6) |
|  | Gloves for delivery | 423(92.5) | 784(97.5) |
|  | Ambulance or other emergency transport | 449(47.7) | 655(81.5) |
|  | Water soap or hand rub | 340(74.3) | 774(96.2) |
|  | Newborn scale | 411(74.3) | 757(94.1) |
| Medicines and commodities | Antibiotic eye drops/ointments | 181(39.5) | 62(7.8) |
|  | Injectable uterotonic | 403(88.2) | 780(97.0) |
|  | Parenteral antibiotics | 187(40.9) | 531(66.1) |
|  | Injectable magnesium sulfate | 330(72.2) | 568(70.7) |
|  | IV solution with infusion set | 413(90.3) | 782(97.2) |
|  | Skin disinfectants | 434(94.8) | 789(98.2) |

*includes national family planning guidelines and other family planning guidelines

**includes national medical standard volume III, national guideline for ANC, and other guidelines for ANC

**health facilities having valid urine dip stick kits for urine protein
